# Dr. Kay A. Smith

**Published:** 1914-09

**Authors:** 


Dr. Kay A. Smith, Buffalo, 1880, died at Bradford, Pa., April 1, 1914, aged 63. (Note· The Buffalo Medical Journal endeavors to publish obituaries of all physicians resident of western N. Y. or graduated from western N. Y. schools or otherwise affiliated with this district. Of the 24 alumni of the University of Buffalo who died during the fiscal year just ended, the names of the above three were omitted. Both as a matter of respect for the dead and for statistic purposes, we ask the co-operation of our readers in securing full and prompt death notices.)

